# Relationship Between the Product of Pre‐Treatment Neutrophil and Monocyte Counts and Clinical Outcomes in Rectal Cancer With Suspected Lateral Lymph Node Metastasis

**DOI:** 10.1002/ags3.70212

**Published:** 2026-03-08

**Authors:** Takayoshi Sasaki, Shinya Abe, Hiroaki Nozawa, Kazuhito Sasaki, Koji Murono, Shigenobu Emoto, Yuichiro Yokoyama, Yuzo Nagai, Yuzo Harada, Soichiro Ishihara

**Affiliations:** ^1^ Department of Surgical Oncology The University of Tokyo Tokyo Japan; ^2^ Department of Surgery The Fraternity Memorial Hospital Tokyo Japan

**Keywords:** chemoradiotherapy, lateral lymph node, lower rectal cancer, monocyte, neutrophil

## Abstract

**Aim:**

A novel systemic inflammatory response marker, the neutrophil × monocyte value (NM value), has been identified as a negative predictive factor for responses to chemoradiotherapy in rectal cancer. However, the clinical implications of the NM value remain unknown.

**Methods:**

This study reviewed 352 patients with rectal cancer who received preoperative chemoradiotherapy between 2003 and 2023. The cut‐off value for the pre‐treatment NM value was established at the median. A pre‐treatment size of lateral lymph node (LLN) ≥ 8 mm was defined as clinical LLN metastasis. The prognostic significance of the NM value was evaluated.

**Results:**

The cut‐off NM value was 1 100 000; 179 patients had an NM value ≥ 1 100 000. Ninety‐nine patients had LLN ≥ 8 mm. Disease‐free survival (DFS) was significantly shorter in patients with an NM value ≥ 1 100 000 than in those with an NM value < 1 100 000. DFS and overall survival (OS) were shorter in patients with LLN ≥ 8 mm than in those with LLN < 8 mm. The LLN size and NM value were identified as independent prognostic factors for DFS (LLN size—hazard ratio (HR): 1.51, *p* = 0.04, NM value—HR: 1.53, *p* = 0.04). Patients with LLN ≥ 8 mm and an NM value ≥ 1 100 000 had the poorest prognosis among the populations compared by the combination of LLN size and NM value.

**Conclusions:**

The pre‐treatment NM value is an independent prognostic factor associated with DFS. In patients with LLN ≥ 8 mm, a high NM value may be a significant indicator of a poor prognosis.

## Abstract 2

The neutrophil × monocyte value (NM value) before preoperative chemoradiotherapy is useful as a predictor of disease‐free survival for lower rectal cancer. In patients with lateral lymph node size ≥ 8 mm on computed tomography, a high NM value may be a significant indicator of a poor prognosis.

**Abbreviations**
CI: confidence interval
CRT: chemoradiotherapy
CT: computed tomography
DFS: disease‐free survival
HR: hazard ratio
IL‐10: interleukin‐10
IQR: interquartile range
LAR: low anterior rectal resection
LLN: lateral lymph node
LLND: lateral lymph node dissection
MDSC: myeloid‐derived suppressor cell
NM value: neutrophil × monocyte value
OS: overall survival
pCR: pathological complete response
TGF‐β: transforming growth factor‐β
TME: total mesorectal excision
TNT: total neoadjuvant therapy

## 1 Introduction

Preoperative chemoradiotherapy (CRT) prior to total mesorectal excision (TME) is widely performed to prevent local recurrence in patients with advanced lower rectal cancer. Novel treatment concepts for lower rectal cancer that promote organ preservation and avoid radical surgery, such as total neoadjuvant therapy (TNT) and the watch‐and‐wait strategy as upfront management, have recently emerged [1, 2, 3]. Predictions of the outcomes of patients requiring preoperative CRT before treatment are useful for informing treatment strategies and selecting chemotherapy regimens.

Various systemic inflammatory response markers have recently gained attention as indicators of prognosis and treatment efficacy in cancer patients. These markers reflect tumor progression and patient‐related factors, including the systemic inflammatory and immune status, and were previously shown to be useful for treatment selection and prognostication. Previous studies suggested that various systemic inflammatory response markers obtained from pre‐treatment blood tests correlated with treatment responses and the prognosis of locally advanced rectal cancer patients receiving preoperative CRT [4, 5, 6, 7, 8, 9]. These findings may be attributed to the modulation of systemic immune responses by preoperative CRT in addition to its local therapeutic effects on tumors [6, 8, 10]. Sawada et al. compared multiple systemic inflammatory response markers and reported that the neutrophil × monocyte (NM) value may be the most useful for predicting CRT responses in lower rectal cancer patients [11]. A good CRT response has been identified as a favorable prognostic indicator in advanced rectal cancer [12, 13]. Therefore, we hypothesize that the NM value has potential as a promising prognostic marker.

Lateral lymph node (LLN) metastasis is also one of the poor prognostic factors for lower rectal cancer, and lateral lymph node dissection (LLND) in conjunction with TME has traditionally been performed in Japan as the standard surgical procedure [14]. Recent strategies involve a pre‐treatment evaluation of clinical LLN metastasis, with the combination of preoperative CRT and selective LLND now being widely practiced. However, current evidence indicates that this approach primarily contributes to local recurrence control rather than directly improving the long‐term prognosis of patients [15, 16, 17]. Therefore, some patients with clinical LLN metastasis may have a systemic disease phase and require more aggressive therapy. We also hypothesized that the NM value may better reflect the host anticancer immune status, leading to long‐term outcomes, particularly in patients with clinical LLN metastasis.

Lymph node enlargement on imaging studies is one indicator of the presence of LLN metastasis [18, 19, 20, 21, 22]. Therefore, the present study investigated the relationship between the prognosis of patients with locally advanced rectal cancer and NM values alone or the combination of pre‐treatment lymph node enlargement and NM values.

## 2 Patients and Methods

### 2.1 Study Population

We herein retrospectively analyzed 352 patients with clinical stage II‐III middle and lower rectal adenocarcinoma cancer who had received preoperative CRT and then underwent curative intent resection at the University of Tokyo Hospital between October 2003 and March 2023. Staging was evaluated using computed tomography (CT) of the chest to pelvis and magnetic resonance imaging of the pelvic region. The present study was approved by the Institutional Ethics Committees of the University of Tokyo (No. 3252‐[15]). Informed consent was obtained through an opt‐out method on the website (http://all‐1su.umin.jp/custom8.html).

### 2.2 Systemic Inflammatory Response Markers

All patients underwent blood tests before preoperative CRT, which confirmed white blood cell counts and their fractions. In the present study, we focused on the product of neutrophil and monocyte counts as an indicator of systemic inflammatory response markers, included it in the analysis, and abbreviated it as the NM value [11].

### 2.3 Treatment

All patients underwent radical resection after preoperative CRT. Radiation therapy was administered at 55 Gy/25 doses/5 weeks or 50.4 Gy/28 doses/5 weeks to the entire pelvis. Concurrent chemotherapy included oral tegafur/uracil + leucovorin with or without irinotecan, oral tegafur/gimeracil/oteracil with oxaliplatin, or the continuous infusion of 5‐fluorouracil. Radical resection was typically performed 6–8 weeks after the completion of CRT.

The following surgical procedures were conducted according to the location and extent of the tumor: low anterior rectal resection (LAR), intersphincteric resection, Hartmann's procedure, abdominoperineal resection, and total pelvic exenteration. LLND was performed selectively when LLN with a long‐axis diameter ≥ 8 mm was observed on pre‐treatment CT images [18].

### 2.4 Evaluation of Clinical and Pathological Features

Clinical data, such as age, sex, physical parameters, distance from the lower tumor margin to the anal verge, the clinical TNM classification, the pre‐treatment white blood cell count and its fractions, the concurrent chemotherapy regimen of CRT, the surgical procedure, histopathology results, and outcomes, were reviewed from electronically stored medical records. Blood tests were performed on all patients as pre‐treatment data within 2–3 weeks prior to the initiation of CRT. LLNs were evaluated using CT, and those with a long‐axis diameter ≥ 8 mm were considered to be clinical metastases [18]. All resected specimens were diagnosed with a histopathological classification according to the American Joint Committee on Cancer classification [23].

### 2.5 Patient Follow‐Up

Postoperative surveillance was conducted according to the Japanese Society for Cancer of the Colon and Rectum guidelines [14]. Blood tests were performed every 3 months, CT every 6 months to detect distant metastases, and a colonoscopy every year. Recurrence was defined as local recurrence or distant metastasis. Depending on the results of histopathological examinations on surgical specimens and the patient's condition, adjuvant chemotherapy was recommended for 6 months after surgery [14]. Positive pathology results in cases of LLND or the recurrence of LLN metastasis without dissection were considered to be positive for LLN metastasis.

### 2.6 Statistical Analysis

Survival curves were analyzed using the Kaplan–Meier method, with the Log‐rank test being applied to compare survival curves. Disease‐free survival (DFS) was defined as the time from the date of surgery to the date on which recurrence, either local or distant, was first identified, secondary cancer was detected, or death from any cause occurred. Overall survival (OS) was defined as the time from the date of surgery to the date of death from any cause. Univariate and multivariate analyses of prognostic factors were performed using the NM value and the following pre‐treatment parameters: sex, age, BMI, smoking history, the Charlson Comorbidity Index, CEA level, tumor distance from the anal verge, clinical T stage, clinical mesorectal lymph node metastasis, pathological type, preoperative chemotherapy regimen, and LLN size, which have been proposed as prognostic factors [24, 25].

Statistical analyses were conducted with JMP Pro 17 (SAS Institute Inc., Cary, NC, USA), and *p* < 0.05 was considered to be significant.

## 3 Results

### 3.1 Patient Characteristics

Patient characteristics are shown in Table 1. CRT and radical surgery were performed on 352 patients, including 224 (63.6%) male and 128 (36.4%) female patients, with a median age of 64 years (interquartile range (IQR): 57–71 years). The median maximum diameter of pre‐treatment LLN was 3.9 mm (IQR: 2.9–5.9), and 99 patients (28.1%) had LLN ≥ 8 mm. The most frequent concurrent chemotherapy regimen with radiation therapy was tegafur/uracil + leucovorin in 254 patients (72.2%). Laparoscopic surgery (125 cases, 35.5%) and robotic surgery (111 cases, 31.5%) accounted for more than half of the surgical procedures performed, with LAR (212 cases, 60.2%) being the most common. LLND was performed bilaterally in 16 patients (4.5%) and unilaterally in 41 patients (12.5%). In 27 patients (7.7%), LLN metastasis was detected on histopathology. After surgery, 108 patients (30.7%) received adjuvant chemotherapy. The final follow‐up date was December 27, 2023, and the median follow‐up duration was 67 months (IQR: 41–109 months).

**TABLE 1:** Patient characteristics.

Variables	Overall
	352
Sex	
Male	224 (63.6%)
Female	128 (36.4%)
Age (years), median (IQR)	64 (57–71)
BMI, median (IQR)	23.0 (20.3–24.1)
Tumor location from AV (cm), median (IQR)	4 (3–6)
Clinical T stage	
≤ 3	315 (89.5%)
4	37 (10.5%)
Clinical mesorectal lymph node metastasis, present	157 (44.6%)
Clinical lateral lymph node metastasis, present^a^	99 (28.1%)
Maximum lateral lymph node size (mm), median (IQR)	3.9 (2.9–5.9)
Pathological type	
Well/moderately	329 (93.5%)
Others	23 (6.5%)
Pathological T stage	
0 (CR)	38 (10.8%)
Tis	9 (2.6%)
1	26 (7.4%)
2	102 (29.0%)
3	156 (44.3%)
4	21 (6.0%)
Pathological mesorectal lymph node metastasis, present	82 (23.3%)
Pathological lateral lymph node metastasis, present	27 (7.7%)
Lymphatic invasion, present	31 (8.8%)
Venous invasion, present	152 (43.2%)
Preoperative chemotherapy regimen	
Tegafur/uracil+leucovorin	254 (72.2%)
Tegafur/uracil +leucovorin+ irinotecan	80 (22.8%)
5‐Fluorouracil	8 (2.3%)
Tegafur/gimeracil/oteracil	4 (1.1%)
Tegafur/gimeracil/oteracil + oxaliplatin	6 (1.7%)
Type of surgery	
Open surgery	116 (33.0%)
Laparoscopic surgery	125 (35.5%)
Robotic surgery	111 (31.5%)
Surgical procedure	
Low anterior resection	212 (60.2%)
Intersphincteric resection	48 (13.6%)
Abdominoperineal resection	83 (23.6%)
Hartmann's procedure	5 (1.4%)
Total pelvic exenteration	4 (1.1%)
Lateral lymph node dissection	
None	295 (83.8%)
Bilateral	16 (4.5%)
Unilateral	41 (11.6%)
Adjuvant chemotherapy regimen	
None	244 (69.3%)
Capecitabine	8 (2.3%)
Capecitabine + oxaliplatin	44 (12.5%)
Tegafur/uracil+leucovorin	45 (12.8%)
Folinic acid + fluorouracil + oxaliplatin	5 (1.4%)
Folinic acid + fluorouracil + oxaliplatin + bevacizumab	1 (0.3%)
Tegafur/gimeracil/oteracil	5 (1.4%)
Postoperative follow‐up duration (months), median (IQR)	67 (41–109)

Abbreviations: AV; anal verge, BMI; body mass index, CI; confidence interval, HR; hazard ratio.

^a^ The clinical lymph node metastasis was defined as having a long‐axis diameter ≥ 8 mm in the pre‐treatment lateral lymph nodes.

### 3.2 Cut‐Off for the NM Value and Evaluation of LLN Sizes

**FIGURE 1:**
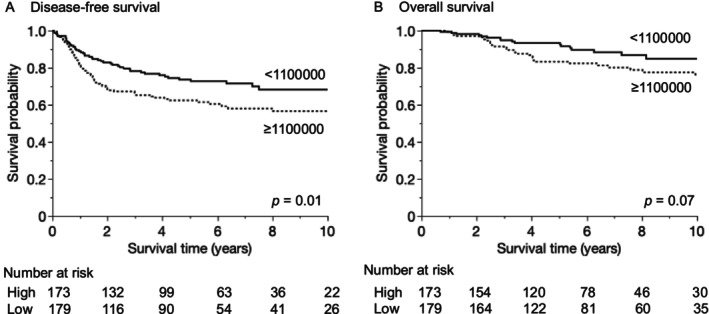
Relationship between DFS (A) and OS (B) according to NM values. DFS is significantly stratified by the NM value (5‐year DFS rate for the NM value < 1 100 000 73.6%, NM value ≥ 1 100 000 62.4%), whereas OS is not (5‐year OS rate for the NM value < 1 100 000 93.4%, NM value ≥ 1 100 000 83.3%).

The cut‐off for the NM value was a median of 1 100 000. Upon performing a receiver operating characteristic (ROC) analysis of NM values for both DFS and OS, we found that Youden's Index was higher for the median value of 11 000 000 than for the mean value or previously reported cut‐off value [11] (DFS—sensitivity: 52.3%, specificity: 62.0%, OS—sensitivity: 55.3%, specificity: 60.0%, Table S1A,B). The relationship between the NM value and DFS or OS is shown in Figure 1A,B. The NM value clearly discriminated DFS rates (5‐year DFS rate: 73.6% vs. 62.4%; *p* = 0.01, Figure 1A) and slightly discriminated OS rates (5‐year OS rate: 93.4% vs. 83.3%; *p* = 0.07, Figure 1B).

**FIGURE 2:**
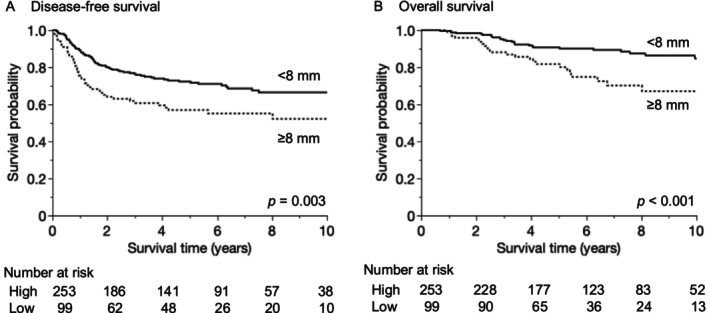
Relationship between DFS (A) and OS (B) according to a lateral lymph node (LLN) size of 8 mm. DFS and OS are both stratified by LLN of 8 mm (5‐year DFS rate for LLN < 8 mm 72.3%, LLN ≥ 8 mm 57.0%, 5‐year OS rate for LLN < 8 mm, 90.7%, LLN ≥ 8 mm 81.7%).

In the present study, a long‐axis diameter of LLN ≥ 8 mm on pre‐treatment CT was clinically considered to be LLN metastasis. The relationship between the size of LLN and prognosis was then analyzed. Figure 2A shows that the 5‐year DFS rate was significantly lower for patients with pre‐treatment LLN ≥ 8 mm (72.3% vs. 57.0%; *p* = 0.003). In addition, the 5‐year OS rate in patients with pre‐treatment LLN ≥ 8 mm was significantly lower than that in patients with pre‐treatment LLN < 8 mm (90.7% vs. 81.7%; *p* < 0.001, Figure 2B).

### 3.3 Analysis Involving NM Values and LLN Sizes

We then investigated the potential of NM values and LLN sizes as independent prognostic factors. The NM value demonstrated significant prognostic discrimination for DFS (Figure 1A) and was identified as an independent prognostic factor in both univariate and multivariate analyses (HR: 1.53, 95% confidence interval (CI): 1.02–2.28; *p* = 0.04) (Table 2). In contrast, the NM value weakly correlated with OS in the univariate, but not multivariate analysis (Table 3). LLN size was identified as an independent prognostic factor for both OS and DFS (OS—hazard ratio (HR): 2.03, 95% CI: 1.13–3.65; *p* = 0.02, DFS—HR: 1.51, 95% CI: 1.03–2.23; *p* = 0.04) (Tables 2, 3).

**TABLE 2:** Univariate and multivariate analyses for disease‐free survival.

	Disease‐free survival					
	Univariate analysis			Multivariate analysis		
	HR	95% CI	*p*	HR	95% CI	*p*
Sex						
Male vs. Female	1.05	0.72–1.53	0.79	0.97	0.62–1.52	0.90
Age (years)						
≥ 60 vs. < 60	1.07	0.73–1.58	0.72	1.00	0.67–1.50	0.99
BMI						
≥ 25 vs. < 25	0.79	0.47–1.32	0.36	0.80	0.47–1.36	0.41
Smoking						
Present vs. Absent	1.06	0.73–1.54	0.76	0.99	0.65–1.53	0.97
Charlson comorbidity index						
≥ 1 vs. 0	1.03	0.70–1.50	0.89	0.89	0.59–1.32	0.55
CEA						
≥ 5 vs. < 5	1.64	1.10–2.43	0.01	1.51	1.00–2.29	0.05
Tumor location from AV (cm)						
< 5 vs. ≥ 5	1.61	1.11–2.34	0.01	1.38	0.94–2.02	0.10
Clinical‐T Stage						
4 vs. ≤ 3	2.27	1.38–3.71	< 0.01	1.67	0.99–2.79	0.05
Clinical mesorectal lymph node metastasis						
Present vs. Absent	1.26	0.87–1.80	0.22	1.09	0.74–1.59	0.67
Pathological type						
Others vs. Well/moderately	1.75	0.94–3.25	0.08	1.58	0.82–3.04	0.17
Preoperative chemotherapy regimen						
Others vs. doublet therapy^a^	0.86	0.56–1.33	0.49	0.93	0.60–1.45	0.76
Lateral lymph node size						
≥ 8 mm vs. < 8 mm	1.77	1.21–2.57	< 0.01	1.51	1.03–2.23	0.04
NM value						
≥ 1 100 000 vs. < 1 100 000	1.63	1.12–2.36	0.01	1.53	1.02–2.28	0.04

Abbreviations: AV; anal verge, BMI; body mass index, CI; confidence interval, HR; hazard ratio.

^a^ “Doublet therapy” was defined as an intensified regimen containing either irinotecan or oxaliplatin, specifically the tegafur/uracil + leucovorin + irinotecan regimen and the tegafur/gimeracil/oteracil + oxaliplatin regimen.

**TABLE 3:** Univariate and multivariate analyses for overall survival.

	Overall survival					
	Univariate analysis			Multivariate analysis		
	HR	95% CI	*p*	HR	95% CI	*p*
Sex						
Male vs. Female	1.22	0.67–2.21	0.51	1.26	0.62–2.56	0.52
Age (years)						
≥ 60 vs. < 60	1.07	0.60–1.92	0.82	1.06	0.57–1.98	0.85
BMI						
≥ 25 vs. < 25	1.06	0.52–2.19	0.87	0.88	0.41–1.93	0.76
Smoking						
Present vs. Absent	1.10	0.62–1.95	0.76	0.98	0.50–1.92	0.96
Charlson comorbidity index						
≥ 1 vs. 0	1.07	0.60–1.93	0.82	0.93	0.50–1.74	0.82
CEA						
≥ 5 vs. < 5	2.00	1.06–3.76	0.03	1.98	1.00–3.91	0.05
Tumor location from AV (cm)						
< 5 vs. ≥ 5	1.98	1.09–3.59	0.02	1.63	0.88–3.05	0.12
Clinical‐T Stage						
4 vs. ≤ 3	3.55	1.76–7.15	< 0.01	2.31	1.11–4.81	0.02
Clinical mesorectal lymph node metastasis						
Present vs. Absent	1.79	1.02–3.12	0.04	1.51	0.84–2.72	0.17
Pathological type						
Others vs. Well/moderately	5.42	2.77–10.62	< 0.01	5.21	2.50–10.88	< 0.01
Preoperative chemotherapy regimen						
Others vs. doublet therapy^a^	0.96	0.45–2.06	0.91	1.09	0.50–2.38	0.83
Lateral lymph node size						
≥ 8 mm vs. < 8 mm	2.63	1.51–4.58	< 0.01	2.03	1.13–3.65	0.02
NM value						
≥ 1 100 000 vs. < 1 100 000	1.69	0.95–3.01	0.07	1.21	0.65–2.26	0.55

Abbreviations: AV; anal verge, BMI; body mass index, CI; confidence interval, HR; hazard ratio.

^a^ “Doublet therapy” was defined as an intensified regimen containing either irinotecan or oxaliplatin, specifically the tegafur/uracil + leucovorin + irinotecan regimen and the tegafur/gimeracil/oteracil + oxaliplatin regimen.

**FIGURE 3:**
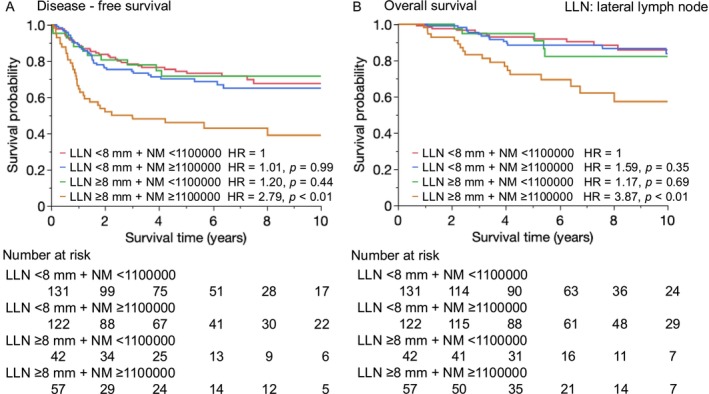
Relationships between DFS (A) and OS (B) and the combination of pre‐treatment lateral lymph node (LLN) sizes < 8 mm and ≥ 8 mm and NM values < 1 100 000 and ≥ 1 100 000. LLN ≥ 8 mm + a high NM value had a significantly worse prognosis for both DFS and OS (DFS hazard ratio for LLN < 8 mm + a low NM value: 2.85, *p* < 0.0001, OS hazard ratio for LLN < 8 mm + a low NM value: 3.82, *p* = 0.0002).

We examined the clinical implications of the combination of a pre‐treatment LLN size < 8 mm or ≥ 8 mm and an NM value < 1 100 000 or ≥ 1 100 000, which were independent prognostic factors for DFS. As shown in Figure 3A,B, patients with pre‐treatment LLN ≥ 8 mm and NM value ≥ 1 100 000 had a significantly poorer long‐term prognosis than those with LLN < 8 mm and NM value < 1 100 000 for both DFS and OS. Furthermore, 50% of these patients developed recurrence within 3 years of surgery (survival probability: 50.1%), and the 5‐year OS rate was significantly lower in this group (survival probability: 72.2%).

## 4 Discussion

The present study demonstrated that the pre‐treatment NM value was a useful predictor of DFS in lower rectal cancer. In addition, the NM value may be a useful prognostic indicator in patients with pre‐treatment LLN ≥ 8 mm. To the best of our knowledge, this is the first study to examine the relationship between the NM value and LLN enlargement as well as its potential as a prognostic factor.

In cancer treatment, new systemic inflammatory response markers have been intensively investigated to predict disease progression more accurately and identify effective therapies. The neutrophil‐to‐lymphocyte ratio, platelet‐to‐lymphocyte ratio, and monocyte‐to‐lymphocyte ratio have been identified as useful prognostic indicators for rectal cancer [4, 5, 6, 7, 8, 9]. Nevertheless, few studies have investigated the prognostic significance of the NM value, a combination of neutrophils and monocytes. Myeloid‐derived suppressor cells (MDSCs) involve both neutrophils and monocytes. MDSCs modulate the tumor microenvironment by suppressing the activity of immune effector cells, such as CD8+ T cells. They accumulate in tumors and release various immunosuppressive factors, including reactive oxygen species, nitric oxide, and immunosuppressive cytokines, including interleukin (IL)‐10 and transforming growth factor (TGF)‐β, which collectively inhibit T cell functions and promote immune evasion. This immunosuppressive activity of MDSCs contributes to tumor progression and growth by preventing effective antitumor immune responses [26, 27]. MDSCs consist of two main subpopulations: polymorphonuclear MDSCs (PMN‐MDSCs) and monocytic MDSCs (M‐MDSCs), which are related to neutrophil and monocyte differentiation, respectively. In cancer patients, PMN‐MDSCs in peripheral blood have been suggested to represent pathologically activated neutrophils that play an important role in regulating immune responses [27, 28]. M2‐like macrophages contribute to immunosuppression in the tumor microenvironment by secreting anti‐inflammatory cytokines, such as IL‐10 and TGF‐β, which suppress antitumor immune responses and promote tumor progression. A previous study demonstrated that M‐MDSCs induced monocytes to differentiate into M2‐like macrophages, further enhancing the immunosuppressive environment [26].

Neutrophils and monocytes are both related to MDSCs and are considered to play roles in the suppression of immune responses within the tumor microenvironment. In the present study, a significantly higher percentage of pathological T4 cases was observed in the group with elevated NM values (Tables S1 and S2), which indicates that the NM value reflects local immune responses in tumors. Collectively, these results suggest that elevated NM values impact the host's antitumor immunity. Therefore, an increase in the recurrence rate is considered to be directly affected by residual micrometastatic cancer cells after surgery, which is supported by the significant difference observed in DFS in the present study (Figure 1A, Table 2). In contrast, the NM value did not emerge as an independent prognostic factor for OS (Table 3). This may be due to variations in treatment strategies depending on the recurrence pattern, with factors such as the resectability of recurrent lesions being among the most significant contributor to survival outcomes [29]. The NM value did not correlate with the pathological complete response (pCR) rate following CRT (Table S2). Although a favorable local tumor response to CRT is anticipated to contribute to an improved prognosis [12, 13], recent findings suggest that the pCR status does not necessarily correlate with survival outcomes [30, 31, 32]. Although pCR reflects local therapeutic efficacy, the impact of CRT on system immune responses must also be considered [10]. Pre‐treatment systemic inflammatory markers have been suggested to reflect the host's immune status and serve as useful prognostic factors in patients receiving CRT [6, 7, 8]. Long‐term outcomes regarding recurrence in the present study support the hypothesis that the pre‐treatment immune status plays a more prominent role in patients receiving CRT. However, the clinical significance of the NM value for the prognosis of rectal cancer patients not receiving CRT, or colon cancer patients, remains unclear and, thus, warrants further investigation.

The high NM value population was significantly more prevalent in males and smokers (Table S2). Smoking induces elevations in neutrophils and monocytes [33, 34], which are attributed to chronic inflammation triggered by inflammatory cytokines, including IL‐6 and TNF‐α. The significantly elevated NM values observed in smokers may be attributed to these inflammatory mechanisms. Furthermore, in the population examined in the present study, males outnumbered females (Table 1), and the percentage of smokers among males was 78.1%. Consequently, the number of male smokers exceeded the total number of females, which may explain the significantly higher NM values in males. However, neither sex nor the smoking status emerged as significant prognostic factors in univariate analyses (Tables 2 and 3). Additionally, in stratified analyses of long‐term prognosis by sex, male and female patients with high NM values and LLN ≥ 8 mm both had poorer prognoses (Figure S1).

The present results showed that patients with LLN enlargement before CRT in the present study had a poor prognosis (Figure 2), which is consistent with previous findings [15, 35]. Furthermore, the integration of NM values, which likely reflect antitumor immunity, with the LLN status allowed for clearer prognostic stratification (Figure 3). In the LLN < 8 mm group, most cases lacked metastatic involvement, likely representing a clinically low‐risk cohort with less advanced disease. In this population, the treatment effects of CRT + surgery were more likely to be reflected in the prognosis of patients, and we considered differences in host antitumor immunity distinguished by NM values to become more difficult to discern. In contrast, the LLN ≥ 8 mm group may have included a higher percentage of cases with actual LLN metastasis, suggesting a more advanced cancer status. In this population, it is hypothesized that although CRT and surgery impact treatment outcomes, the NM value, reflecting antitumor immunity, may also affect the prognosis of patients, potentially leading to significant differences in survival outcomes. These results highlight the utility of the NM value in predicting the outcomes of patients with larger LLN, which often indicate more advanced disease stages and necessitate precise management decisions. This high‐risk population may benefit from more intensive preoperative treatment strategies, such as TNT, to improve their prognosis by addressing systemic tumor treatment and immune dysregulation reflected by the NM value.

This study has several limitations that need to be addressed. This was a retrospective analysis conducted at a single institution. In the present study, not all cases underwent LLND, which may have affected the results obtained. We also used a cut‐off value of 8 mm for the pre‐treatment LLN size, a criterion that remains debated in the literature. The prediction of LLN metastasis is currently assessed through various imaging modalities, including lymph node size, morphology, and CT attenuation values; however, there is still no established consensus [19, 20, 21, 22]. At our institution, an analysis of LLN sizes has shown that pre‐treatment LLN ≥ 8 mm is the optimal criterion for LLND, with a sensitivity of 92.3% and specificity of 78.7% [18]. Before this previously reported study, no clear criteria had been established for performing LLND. In comparison with previous studies, this cut‐off value is considered to be useful and, thus, we set LLN ≥ 8 mm as the criterion in this analysis. In addition to variability in the indication criteria for LLND, this cohort followed patients over a 20‐year period, which inherently introduced a bias because of changes in treatment regimens and surgical techniques over time. Since 2019, the percentage of patients receiving a CRT regimen consisting of tegafur/uracil + leucovorin + irinotecan has increased at our institution, constituting a temporal bias. Furthermore, we herein utilized the NM value as a systemic inflammatory response marker; however, no specific cut‐off value has been established. Sawada et al. defined the cut‐off of NM value on the basis of the tumor regression grade, suggesting that the NM value serves as a predictor of CRT responsiveness and prognosis. However, the present analysis did not reveal a correlation between NM values and the pCR rate (Table S2). Additionally, to identify an appropriate cut‐off value, we conducted ROC analyses of both OS and DFS, comparing the mean value, median value, and the value established by Sawada et al. The ROC analysis of DFS yielded an AUC of 0.58 at an NM value = 1 100 000, which coincided with the median value (Table S1A), whereas the analysis of OS resulted in an AUC of 0.60 at an NM value = 1 100 000 (Table S1B). Youden's Index indicated that the median value was higher for both OS and DFS than for the mean value and the value reported by Sawada et al. The present study aimed to elucidate whether high versus low NM values were associated with prognosis, and, thus, we selected the median value as our cut‐off. However, it is important to note that an optimal cut‐off value was not definitively established, which is a major limitation of this study. Further accumulation of research using systemic inflammatory response markers is needed to select the optimal NM value. Additionally, the timing of blood sample collection used in analyses and the initiation of treatment were not entirely consistent, which may have affected the results obtained.

Despite the limited predictive power of the NM value for long‐term outcomes, its potential prognostic significance in patients with enlarged LLNs provides novel insights into patient stratification and treatment strategies. Further prospective data and detailed analyses are necessary to confirm this approach and optimize treatment strategies.

## 5 Conclusion

In patients with lower rectal cancer, the pre‐treatment NM value was identified as an independent prognostic factor associated with DFS. Furthermore, in patients with LLN ≥ 8 mm, a high NM value appears to be a significant indicator of a poor prognosis.

## Author Contributions

**Takayoshi Sasaki:** conceptualization, methodology, validation, formal analysis, investigation, data curation, writing – original draft, visualization. **Shinya Abe:** conceptualization, methodology, validation, investigation, data curation, writing – review and editing. **Hiroaki Nozawa:** writing – review and editing. **Kazuhito Sasaki:** writing – review and editing. **Koji Murono:** writing – review and editing. **Shigenobu Emoto:** writing – review and editing. **Yuichiro Yokoyama:** writing – review and editing. **Yuzo Nagai:** writing – review and editing. **Yuzo Harada:** writing – review and editing. **Soichiro Ishihara:** writing – review and editing, supervision.

## Funding

The authors have nothing to report.

## Ethics Statement

The present study was approved by the Institutional Ethics Committees of the University of Tokyo (No. 3252‐ [15]).

## Conflicts of Interest

The authors declare no conflicts of interest.

## Supporting information


**Figure S1:** Relationships between DFS and OS and the combination of pre‐treatment lateral lymph node (LLN) sizes < 8 mm and ≥ 8 mm and NM values < 1 100 000 and ≥ 1 100 000 by sex. In females, LLN ≥ 8 mm + a high NM value were significantly worse prognostic factors for both DFS and OS (DFS hazard ratio for LLN < 8 mm + a low NM value: 7.51, *p* < 0.01, OS hazard ratio for LLN < 8 mm + a low NM value: 4.73, *p* = 0.02) (A). In males, LLN ≥ 8 mm + a high NM value were significantly worse prognostic factors for both DFS and OS (DFS hazard ratio for LLN < 8 mm + a low NM value: 1.78, *p* = 0.05, OS hazard ratio for LLN < 8 mm + a low NM value: 3.58, *p* < 0.01).

**Table S1:** Comparison of the sensitivity and specificity of NM values in receiver operating characteristic analyses.

**Table S2:** Comparisons of patients with high/low NM values and lateral lymph node < 8 mm ≥ 8 mm.
