# Supplementary material for: Relationship Between the Product of Pre‐Treatment Neutrophil and Monocyte Counts and Clinical Outcomes in Rectal Cancer With Suspected Lateral Lymph Node Metastasis
Source: Ann Gastroenterol Surg. 2026 Mar 8;10(4):1097–106. doi: 10.1002/ags3.70212 (PMC13326825; doi:10.1002/ags3.70212)
Supplement: Supplementary file 1 — Figure S1: Relationships between DFS and OS and the combination of pre‐treatment lateral lymph node (LLN) sizes < 8 mm and ≥ 8 mm and NM values < 1 100 000 and ≥ 1 100 000 by sex. In females, LLN ≥ 8 mm + a high NM value were significantly worse prognostic factors for both DFS and OS (DFS hazard ratio for LLN < 8 mm + a low NM value: 7.51, p < 0.01, OS hazard ratio for LLN < 8 mm + a low NM value: 4.73, p = 0.02) (A). In males, LLN ≥ 8 mm + a high NM value were significantly worse prognostic factors for both DFS and OS (DFS hazard ratio for LLN < 8 mm + a low NM value: 1.78, p = 0.05, OS hazard ratio for LLN < 8 mm + a low NM value: 3.58, p < 0.01). [file AGS3-10-1097-s003.pdf]

A

Female

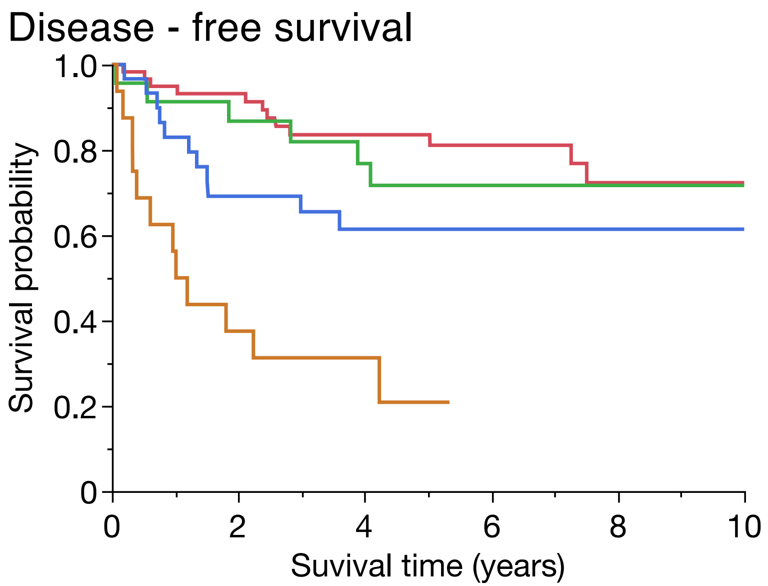

- LLN <8 mm + NM <1100000 HR = 1
- LLN <8 mm + NM ≥1100000 HR = 2.18, $p = 0.06$
- LLN ≥8 mm + NM <1100000 HR = 1.34, $p = 0.56$
- LLN ≥8 mm + NM ≥1100000 HR = 7.51, $p < 0.01$

Number at risk

LLN <8 mm + NM <1100000					
59	51	39	24	15	10
LLN <8 mm + NM ≥1100000					
30	21	14	11	10	10
LLN ≥8 mm + NM <1100000					
23	20	16	9	6	4
LLN ≥8 mm + NM ≥1100000					
16	7	4			

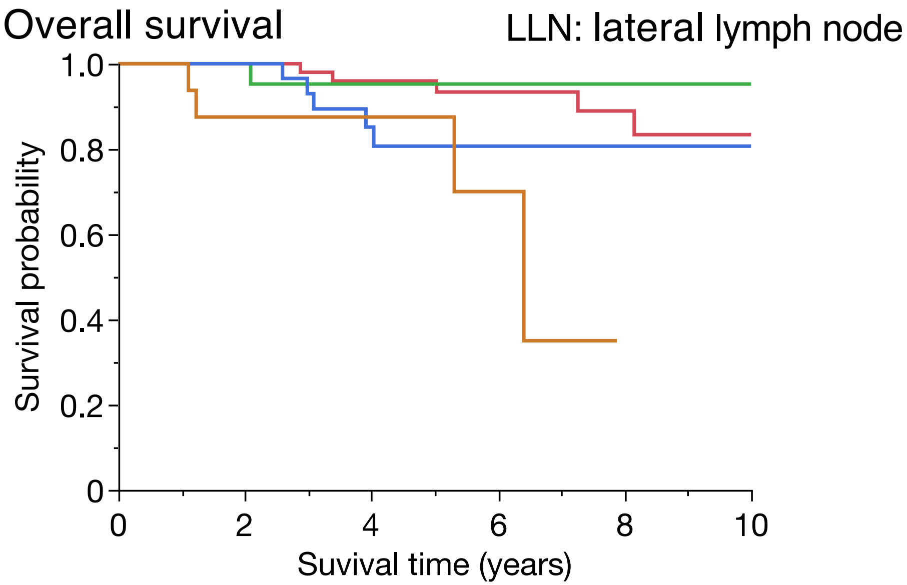

- LLN <8 mm + NM <1100000 HR = 1
- LLN <8 mm + NM ≥1100000 HR = 1.70, $p = 0.40$
- LLN ≥8 mm + NM <1100000 HR = 1.05, $p = 0.96$
- LLN ≥8 mm + NM ≥1100000 HR = 4.73, $p = 0.02$

Number at risk

LLN <8 mm + NM <1100000					
58	54	43	26	17	11
LLN <8 mm + NM ≥1100000					
30	30	20	15	14	12
LLN ≥8 mm + NM <1100000					
23	22	18	11	7	5
LLN ≥8 mm + NM ≥1100000					
16	14	10	4		

B

Male

Disease - free survival

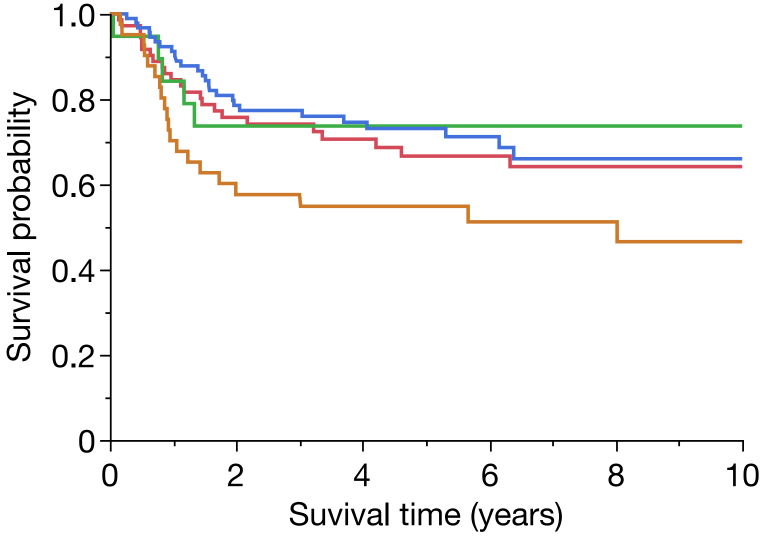

– LLN <8 mm + NM <1100000 HR = 1
– LLN <8 mm + NM ≥1100000 HR = 0.86, $p = 0.59$
– LLN ≥8 mm + NM <1100000 HR = 0.85, $p = 0.73$
– LLN ≥8 mm + NM ≥1100000 HR = 1.78, $p = 0.05$

Number at risk

LLN <8 mm + NM <1100000					
72	49	37	28	14	8
LLN <8 mm + NM ≥1100000					
92	68	54	31	21	13
LLN ≥8 mm + NM <1100000					
19	15	10	5	4	3
LLN ≥8 mm + NM ≥1100000					
41	23	21	14	12	5

Overall survival

LLN: lateral lymph node

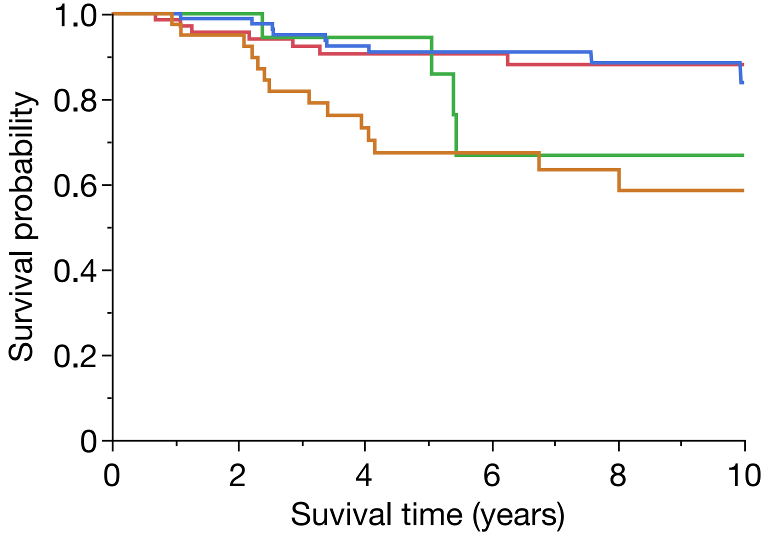

– LLN <8 mm + NM <1100000 HR = 1
– LLN <8 mm + NM ≥1100000 HR = 0.93, $p = 0.90$
– LLN ≥8 mm + NM <1100000 HR = 2.19, $p = 0.21$
– LLN ≥8 mm + NM ≥1100000 HR = 3.58, $p < 0.01$

Number at risk

LLN <8 mm + NM <1100000					
72	61	48	38	20	14
LLN <8 mm + NM ≥1100000					
92	86	69	47	35	18
LLN ≥8 mm + NM <1100000					
19	19	14	6	5	3
LLN ≥8 mm + NM ≥1100000					
41	37	26	18	14	7
