# Supplementary material for: Relationship Between the Product of Pre‐Treatment Neutrophil and Monocyte Counts and Clinical Outcomes in Rectal Cancer With Suspected Lateral Lymph Node Metastasis
Source: Ann Gastroenterol Surg. 2026 Mar 8;10(4):1097–106. doi: 10.1002/ags3.70212 (PMC13326825; doi:10.1002/ags3.70212)
Supplement: Supplementary file 2 — Table S1: Comparison of the sensitivity and specificity of NM values in receiver operating characteristic analyses. [file AGS3-10-1097-s001.docx]

Supplementary Table 1 Comparison of the sensitivity and specificity of NM values in receiver operating characteristic analyses

A. Sensitivity and specificity of NM values for disease-free survival

|  | Cut-off value | Sensitivity (%) | Specificity (%) | Youden’s Index | AUC |
| --- | --- | --- | --- | --- | --- |
| The value calculated from the ROC analysis | 1100000 | 55.3 | 60.0 | 0.153 | 0.58 |
| Median value | 1100000 | 55.3 | 60.0 | 0.153 |  |
| Mean value | 1304816 | 66.0 | 46.2 | 0.122 |  |
| The value proposed by Sawada et al. | 1440000 | 71.9 | 41.0 | 0.129 |  |

B. Sensitivity and specificity of NM values for overall survival

|  | Cut-off value | Sensitivity (%) | Specificity (%) | Youden’s Index | AUC |
| --- | --- | --- | --- | --- | --- |
| The value calculated from the ROC analysis | 1000000 | 44.0 | 74.0 | 0.180 | 0.60 |
| Median value | 1100000 | 52.3 | 62.0 | 0.143 |  |
| Mean value | 1304816 | 63.6 | 48.0 | 0.116 |  |
| The value proposed by Sawada et al. | 1440000 | 69.5 | 44.0 | 0.135 |  |
