# Supplementary material for: Relationship Between the Product of Pre‐Treatment Neutrophil and Monocyte Counts and Clinical Outcomes in Rectal Cancer With Suspected Lateral Lymph Node Metastasis
Source: Ann Gastroenterol Surg. 2026 Mar 8;10(4):1097–106. doi: 10.1002/ags3.70212 (PMC13326825; doi:10.1002/ags3.70212)
Supplement: Supplementary file 3 — Table S2: Comparisons of patients with high/low NM values and lateral lymph node < 8 mm ≥ 8 mm. [file AGS3-10-1097-s002.docx]

Supplementary Table 2 Comparisons of patients with high/low NM values and lateral lymph node <8 mm ≥8 mm

|  |  | All | | *p* value | LLN <8 mm | | *p* value | LLN ≥8 mm | | *p* value |
| --- | --- | --- | --- | --- | --- | --- | --- | --- | --- | --- |
|  |  | *NM  High n = 179 | *NM  Low n = 173 |  | *NM  High n = 122 | *NM  Low n = 131 |  | *NM High n = 57 | *NM Low n = 42 |  |
| Sex | |  |  | < 0.01 |  |  | < 0.01 |  |  | < 0.01 |
|  | Male | 133 (74.3) | 91 (52.6) |  | 92 (75.4) | 72 (55.0) |  | 41 (71.9) | 19 (45.2) |  |
|  | Female | 46 (25.7) | 82 (47.4) |  | 30 (24.6) | 59 (45.0) |  | 16 (28.1) | 23 (54.8) |  |
| Age, years | |  |  | 0.37 |  |  | 0.13 |  |  | 0.01 |
|  | <60 | 58 (32.4) | 64 (37.0) |  | 33 (27.1) | 47 (35.9) |  | 25 (43.9) | 17 (40.5) |  |
|  | ≥60 | 121 (67.6) | 109 (63.0) |  | 89 (73.9) | 84 (64.1) |  | 32 (56.1) | 25 (59.2) |  |
| BMI | |  |  | 0.39 |  |  | 0.20 |  |  | 0.59 |
|  | <25 | 146 (81.6) | 147 (85.0) |  | 100 (82.0) | 115 (87.8) |  | 46 (80.7) | 32 (76.2) |  |
|  | ≥25 | 33 (18.4) | 26 (15.0) |  | 22 (18.0) | 16 (12.2) |  | 11 (19.3) | 10 (23.8) |  |
| Smoking | |  |  | < 0.01 |  |  | < 0.01 |  |  | < 0.01 |
|  | Absent | 52 (29.1) | 85 (49.1) |  | 33 (27.1) | 60 (45.8) |  | 19 (33.3) | 25 (59.5) |  |
|  | Present | 127 (70.9) | 88 (50.9) |  | 89 (72.9) | 54.2) |  | 38 (66.7) | 17 (40.5) |  |
| Clinical T stage | |  |  | 0.01 |  |  | 0.05 |  |  | 0.17 |
|  | ≤3 | 153 (85.5) | 162 (93.6) |  | 107 (87.7) | 124 (94.7) |  | 46 (80.7) | 38 (90.5) |  |
|  | 4 | 26 (14.5) | 11 (6.4) |  | 15 (12.3) | 7 (5.3) |  | 11 (19.3) | 4 (9.5) |  |
| Clinical mesorectal lymph node metastasis | |  |  | 0.50 |  |  | 0.68 |  |  | 0.10 |
|  | Absent | 96 (53.6) | 99 (57.2) |  | 73 (59.8) | 75 (57.3) |  | 23 (40.4) | 24 (57.1) |  |
|  | Present | 83 (46.4) | 74 (42.7) |  | 49 (40.2) | 56 (42.7) |  | 34 (59.6) | 18 (42.9) |  |
| Pathological type | |  |  | 0.32 |  |  | 0.89 |  |  | 0.18 |
|  | Well/moderately | 165 (92.2) | 164 (94.8) |  | 115 (94.3) | 124 (94.7) |  | 50 (87.7) | 40 (95.2) |  |
|  | others | 14 (7.8) | 9 (5.2) |  | 7 (5.7) | 7 (5.3) |  | 7 (12.3) | 2 (4.8) |  |
| Pathological complete response | |  |  | 0.35 |  |  | 0.14 |  |  | 0.39 |
|  | Absent | 160 (89.4) | 149 (86.1) |  | 110 (90.2) | 110 (84.0) |  | 50 (87.7) | 39 (92.9) |  |
|  | Present | 19 (10.6) | 24 (13.9) |  | 12 (9.8) | 21 (16.0) |  | 7 (12.3) | 3 (7.1) |  |
| Pathological T stage | |  |  | < 0.01 |  |  | 0.01 |  |  | < 0.01 |
|  | ≤3 | 160 (89.4) | 171 (98.8) |  | 112 (91.8) | 129 (98.5) |  | 48 (84.2) | 42 (100) |  |
|  | 4 | 19 (10.6) | 2 (1.2) |  | 10 (8.2) | 2 (1.5) |  | 9 (15.8) | 0 (0) |  |
| Pathological mesorectal lymph node metastasis | |  |  | 0.94 |  |  | 0.54 |  |  | 0.34 |
|  | Absent | 137 (76.5) | 133 (76.9) |  | 97 (79.5) | 100 (76.3) |  | 40 (70.2) | 33 (78.6) |  |
|  | Present | 42 (23.5) | 40 (23.1) |  | 25 (20.5) | 31 (23.7) |  | 17 (29.8) | 9 (21.4) |  |
| Pathological lateral lymph node metastasis | |  |  | 0.61 |  |  | 0.10 |  |  | 0.78 |
|  | Absent or unknown** | 164 (91.6) | 161 (93.1) |  | 122 (100) | 129 (98.5) |  | 42 (73.7) | 32 (76.2) |  |
|  | Present | 15 (8.4) | 12 (6.9) |  | 0 (0) | 2 (1.5) |  | 15 (26.3) | 10 (23.8) |  |
| Lymphatic invasion | |  |  | 0.93 |  |  | 0.58 |  |  | 0.49 |
|  | Absent | 163 (91.1) | 158 (91.3) |  | 114 (93.4) | 120 (91.6) |  | 49 (86.0) | 38 (90.5) |  |
|  | Present | 16 (8.9) | 15 (8.7) |  | 8 (6.6) | 11 (8.4) |  | 8 (14.0) | 4 (9.5) |  |
| Venous invasion | |  |  | 0.97 |  |  | 0.54 |  |  | 0.03 |
|  | Absent | 94 (52.5) | 106 (61.3) |  | 67 (54.9) | 77 (58.8) |  | 27 (47.4) | 29 (69.1) |  |
|  | Present | 85 (47.5) | 67 (38.7) |  | 55 (45.1) | 54 (41.2) |  | 30 (52.6) | 13 (30.9) |  |
| Preoperative chemotherapy regimen | |  |  | 0.19 |  |  | 0.75 |  |  | 0.06 |
|  | Doublet therapy*** | 49 (27.4) | 37 (21.4) |  | 30 (24.6) | 30 (22.9) |  | 19 (33.3) | 7 (16.7) |  |
|  | Others | 130 (72.6) | 136 (78.6) |  | 92 (75.4) | 101 (77.1) |  | 38 (66.7) | 35 (83.3) |  |
| Type of surgery | |  |  | 0.02 |  |  | 0.02 |  |  | 0.56 |
|  | Open | 69 (38.6) | 47 (27.2) |  | 51 (41.8) | 36 (27.5) |  | 18 (31.6) | 11 (26.2) |  |
|  | Laparoscopic/robotic | 110 (61.4) | 126 (72.8) |  | 71 (58.2) | 95 (72.5) |  | 39 (68.4) | 31 (73.8) |  |
| Lateral lymph node dissection | |  |  | 0.46 |  |  | 0.60 |  |  | 0.83 |
|  | Absent | 148 (82.7) | 148 (85.6) |  | 121 (99.2) | 129 (98.5) |  | 27 (47.4) | 19 (45.2) |  |
|  | Present | 31 (17.3) | 25 (14.5) |  | 1 (0.8) | 2 (1.5) |  | 30 (52.6) | 23 (54.8) |  |
| Adjuvant chemotherapy | |  |  | 0.10 |  |  | 0.72 |  |  | 0.02 |
|  | Absent | 117 (65.4) | 127 (73.4) |  | 85 (69.7) | 94 (71.8) |  | 32 (56.1) | 33 (78.6) |  |
|  | Present | 62 (34.6) | 46 (26.6) |  | 37 (30.3) | 37 (28.2) |  | 25 (43.9) | 9 (21.4) |  |

BMI: body mass index

*The cut-off value for categorizing the NM value as high or low was set at 1100000.

**In cases on which lateral lymph node dissection was performed, the status was coded as “Absent”, while in cases without dissection, it was coded as “Unknown”.

***“Doublet therapy” was defined as an intensified regimen containing either irinotecan or oxaliplatin, specifically the tegafur/uracil + leucovorin + irinotecan regimen and the tegafur/gimeracil/oteracil + oxaliplatin regimen.
